# TLR2-Dependent Signaling for IL-15 Production Is Essential for the Homeostasis of Intestinal Intraepithelial Lymphocytes

**DOI:** 10.1155/2016/4281865

**Published:** 2016-08-01

**Authors:** Yuan Qiu, Aimin Pu, Hong Zheng, Minqiang Liu, Weigang Chen, Wensheng Wang, Weidong Xiao, Hua Yang

**Affiliations:** ^1^Department of General Surgery of Xinqiao Hospital, The Third Military Medical University, Shapingba, Chongqing 400037, China; ^2^Department of Thoracic Surgery of Xinqiao Hospital, The Third Military Medical University, Shapingba, Chongqing 400037, China

## Abstract

TLR2 signaling is related to colitis and involved in regulation of innate immunity in the intestinal tract, but the mechanisms remain unclear. The aim of this study is to investigate how TLR2 affects differentiation of intraepithelial lymphocytes (IELs) and regulates the susceptibility of colitis. IELs were isolated from the small intestine and colon of mice, respectively. The IEL phenotype, activation, and apoptosis were examined using flow cytometry and RT-PCR. IL-15 expression and IEL location were detected through immunohistochemistry. The experimental colitis was induced by administration of dextran sulfate sodium (DSS). We found that the numbers of CD8*αα* ^+^, CD8*αβ* ^+^, and TCR*γδ* ^+^ IELs were significantly decreased in TLR2-deficient mice and the residual IELs displayed reduced activation and proliferation and increased apoptosis, accompanied with impaired IL-15 expression by intestinal epithelial cells (IECs). Further study showed that TLR2 signaling maintained the expression of IL-15 in IEC via NF-*κ*B activation. Moreover, TLR2-deficient mice were found to be more susceptible to DSS-induced colitis as shown by the increased severity of colitis. Our results demonstrate that IECs contribute to the maintenance of IELs at least partly via TLR2-dependent IL-15 production, which provides a clue that may link IECs to innate immune protection of the host via IELs.

## 1. Introduction

Intestinal epithelial cells (IECs) maintain a fundamental immunoregulatory function that influences the development and homeostasis of mucosal immune cells. Interestingly, this single layer is home to an abundant population of intestinal intraepithelial lymphocytes (IELs). Though primarily comprised of CD8^+^ T cells, the intestinal IELs can be classified into two major subgroups [1]. One group consists of CD4^+^ or CD8*αβ* ^+^TCR*αβ* ^+^ IELs, which are known as type a IELs. Type b IELs express *αβ*TCRs or *γδ*TCRs with a unique coreceptor, CD8*αα*. About 65–75% of small intestinal IELs are type b IELs (CD8*αα* ^+^TCR*αβ* ^+^ and CD8*αα* ^+^TCR*γδ* ^+^) in mice. Most CD8*αα* ^+^ IEL precursors go through a thymic stage of development and complete maturation in the intestine [2]. We and others previously reported that intestinal gut-derived cytokines are particularly important for the homeostatic proliferation and survival of these IELs. For example, specific intestinal IL-7 overexpression (IL-7^vill^ mice) significantly increased the number of type a IELs but did not have much effect on type b cell numbers [3]. Mice lacking the IL-15 system, including IL-15^−/−^, IL-15R*α* ^−/−^, and IL-15R*β* ^−/−^ mice, showed a severe reduction in CD8*αα* ^+^TCR*αβ* ^+^ and TCR*γδ* ^+^ IELs [4, 5], combined with previous evidence showing that cytokines of the common *γ*-chain (*γ*c) family (e.g., IL-7, IL-15) are critical for development of IELs and depend on *γ*c for cellular signaling.

Toll-like receptors (TLRs) are the main pattern recognition receptors (PRRs) that can recognize pathogen-associated molecular patterns derived from a diverse collection of microbes. TLRs are inducible or constitutively expressed in different combinations throughout the whole intestinal tract by a wide variety of cell types, including IECs, myofibroblasts, and immune cells [6, 7]. TLR signaling has been shown to be involved in epithelial cell proliferation, cytokine production, and antimicrobial peptide expression [8]. Of the TLRs, TLR2 is involved in recognizing a wide range of ligands, including peptidoglycan [9], lipoteichoic acid (LTA) [10], and lipoarabinomannan [11]. A major signaling target of the TLR2 is activation of the transcription factor NF-*κ*B, which is shuttled from the cytosol to the nucleus where it initiates expression of pro- and anti-inflammatory cytokines [12]. Indeed, our recent study has shown that the higher level of infection burden in TLR2^−/−^ mice was closely associated with a reduction in proinflammatory cytokines in the liver [13]. Furthermore, polymorphisms in genes encoding TLR2 and NF-*κ*B signaling proteins (NFKB1 and NFKBIA) are associated with risk of inflammatory bowel disease (IBD) [14]. Thus, it is hypothesized that TLR signaling for cytokine expression is involved in maintenance of IELs and protection of intestinal mucosa.

In this report, we found that the numbers of IELs (especially type b IELs) were reduced significantly in TLR2^−/−^ mice and residual IELs displayed reduced activation and proliferation, accompanied with impaired expression of IL-15 in intestinal mucosa. Our results also indicate that deficiency of TLR2 contributes to the high susceptibility of mice to dextran sulfate sodium- (DSS-) induced colitis.

## 2. Methods

### 2.1. Animals

TLR2^−/−^ mice (C57BL/6J background) were kindly provided by Wenyue Xu (Department of Pathogenic Biology, Third Military Medical University, Chongqing, China). Specific pathogen-free wild-type (WT) C57BL/6J mice were purchased from the Laboratory Animal Center of Third Military Medical University. Six- to eight-week-old, pathogen-free, male mice weighing 20 ± 3 g were used. Mice were maintained in temperature-, humidity-, and light-controlled conditions and all studies were performed under the guidelines of the Institutional Animal Care and Use Committee of Third Military Medical University. All experimental protocols were approved by this committee. For lipoteichoic acid (LTA, Invivogen, USA) injection, LTA dissolved in PBS (5 *μ*g/200 *μ*L/mouse) was injected intravenously via the tail vein, and the intestinal samples were collected 24 h later.

### 2.2. DSS-Induced Colitis

Animals received either regular drinking water (control) or drinking water within 3% DSS (molecular weight: 36,000–50,000; MP Biomedicals, Cleveland, USA) for 7 days. Fresh DSS solution was provided every day. The mice were checked each day for morbidity and weight was recorded. Mice were sacrificed ten days after the first DSS administration. The colons were removed and colonic sections were stained with H&E.

### 2.3. Cell Culture

The human colon adenocarcinoma cell line SW480 was purchased from China Center for Type Culture Collection (Beijing, China). The cells were cultured in RPMI1640 supplemented with 10% fetal bovine serum (Gibco, Carlsbad, USA), 100 IU/mL penicillin, and 100 *μ*g/mL streptomycin (Invitrogen, Carlsbad, CA). Cells were incubated in a 5% CO_2_ humidified incubator at 37°C. The culture medium was changed every two days.

### 2.4. IEL Preparation

Intestinal IEL isolation was carried out as described previously [15]. Briefly, small intestine included tissues from the duodenal bulb to the ileocecal junction. Mesenteric fat and Peyer's patches were carefully removed. The small intestine or colon was opened longitudinally, washed in an IEL extraction buffer (1 mM EDTA, 1 mM DTT in PBS), and cut into 5 mm pieces. The pieces were stirred in the same buffer at 37°C for 25 min. The tissue suspension was filtered rapidly through a glass wool column to remove debris and centrifuged at 1500 RPM at 4°C for 5 min. Pelleted cells were suspended in 20 mL 40% isotonic Percoll (GE Healthcare Biosciences, Piscataway, USA) and centrifuged at 2200 RPM at 4°C for 22 min. The supernatant was carefully sucked off, leaving about 5 mL of solution (containing cells). And the pellet was resuspended in 2 mL Tris-NH_4_Cl (heated to 37°C) for red blood cell lysis. The cells were washed twice and resuspended in RPMI1640. The viability of the IELs exceeded 95%, according to trypan blue exclusion staining.

### 2.5. Flow Cytometric Analysis

The IELs were fluorescence-labeled with the following antibodies: CD45-PE, CD45-PerCP-Cy5.5, CD8*α*-APC, CD8*β*-FITC, CD4-PE, TCR*β*-APC, TCR*γδ*-FITC, and CD69-FITC. All antibodies were obtained from eBioscience (San Diego, USA). 2 × 10^5^ cells were suspended in 50 *μ*L staining buffer (eBioscience, San Diego, USA) with saturating amounts of antibodies and incubated for 30 min at 4°C. For BrdU pulse-chase experiments, BrdU dissolved in sterile isotonic saline (10 mg/mL) was injected twice daily (0.1 mg BrdU/g body wt. per d, i.p.), and IELs were collected 24 h later. For detection of BrdU, cells were stained with FITC-conjugated anti-BrdU (BrdU Flow kit; BD Pharmingen, USA), according to the manufacturer's instructions. The apoptotic ratios for the IELs were measured using Annexin-V-FITC/PI Apoptosis Detection Kit (eBioscience, USA) according to the manufacturer's protocol. Data acquisition and analysis were performed with MoFlow (Beckman Coulter, US) and FlowJo (Three Star, Ashland, USA).

### 2.6. Histological Examination and Immunohistochemistry

Specimens for histological examination were fixed in 4% paraformaldehyde for 48 h. Representative sections of colon and jejunum were cut and embedded in paraffin. 5 *μ*m sections were then cut and stained with H&E.

The small-intestinal tissue blocks were mounted on glass slides. The tissue sections were deparaffinised and rehydrated with xylene and graded alcohol. The primary antibodies, anti-CD3 (1 : 700; Abcam, Cambridge, MA), anti-IL-15 (1 : 200; Abcam, Cambridge, MA), and purified rabbit IgG (10 mg/mL, negative control) were incubated overnight at 4°C. Subsequently, the sections were incubated for 60 min with biotinylated goat anti-rabbit IgG (1 : 100; Wuhan Boster, China) for 60 min, followed by incubation with streptavidin-enzyme conjugate (Wuhan Boster, China). Finally, the sections were counterstained with haematoxylin. An expert observer, blinded to the experimental conditions, examined the tissue sections.

### 2.7. Dual-Luciferase Assay

Dual-luciferase assay for NF-*κ*B was performed as described previously [16]. Briefly, twenty-four hours before transfection, 1 × 10^5^ SW480 cells were seeded per well of a 24-well plate. Each pBIIx-luciferase reporter gene plasmid (100 ng, a gift from Dr. Sankar Ghosh's Lab, Yale University) was cotransfected with TK-RL (10 ng, Promega, USA) for normalization and either 25 nmol/L of TLR2-siRNA (sense: 5′-GCCCUCUCUACAAACUUUATT -3′; antisense: 5′-UAAAGUUUGUAGAGAGGGCTT-3′) or negative control oligonucleotide (sense: 5′-UUCUCCGAACGUGUCACGUTT-3′; antisense: 5′-ACGUGACACGUUCGGAGAATT-3′). At 24 h after transfection, the cells were stimulated with TLR2 agonist, LTA (500 ng/mL), for 6 h. Then, cell extracts were obtained using the dual-luciferase assay kit according to the manufacturer's protocol.

### 2.8. Quantitative Real-Time PCR

Total RNA was extracted from isolated IELs using TRIzol (Invitrogen, Carlsbad, CA). RNA was reverse-transcribed into complementary DNA (cDNA) using a SuperScript First-Strand Synthesis System RT-PCR kit (Invitrogen). This cDNA was used as a template for the amplification of KGF, IL-2, IL-10, RegIII*γ*, and *β*-actin. Quantitative PCR was performed by SYBR Premix Ex TaqTM II (TaKaRa, Japan) using an ABI 7500 (Applied Biosystems, USA). The primers selected are as follows: KGF, F: CGCAAATGGATACTGACACG, R: GGGCTGGAACAGTTCACACT; IL-2, F: CCTGGAGCAGCTGTTGATGG, R: CAGAACATGCCGCAGAGGTC; IFN-*γ*, F: TCAAGTGGCATAGATGTGGAAGAA, R: TGGCTCTGCAGGATTTTCATG; RegIII*γ*, F: TTCCTGTCCTCCATGATCAAAA, R: CATCCACCTCTGTTGGGTTCA; IL-7, F: TCTGCTGCCTGTCACATCATCT, R: AAGTTTGGTTCATTATTCGGG; IL-15, F: ATGTTCATCAACACGTCCTGACT, R: GCAGCAGGTGGAGGTACCTTAA; *β*-actin, F: CTTCTTTGCAGCTCCTTCGTT, R: AGGAGTCCTTCTGACCCATTC. The expression of each gene was normalized to *β*-actin expression in the individual samples.

### 2.9. Statistical Analysis

All data are expressed as means ± SD. Differences were analyzed by Student's *t*-test (with 95% confidence interval). *P* values < 0.05 were considered significant.

## 3. Results

### 3.1. TLR2 Deficiency Results in the Loss of IELs

**Figure 1:**
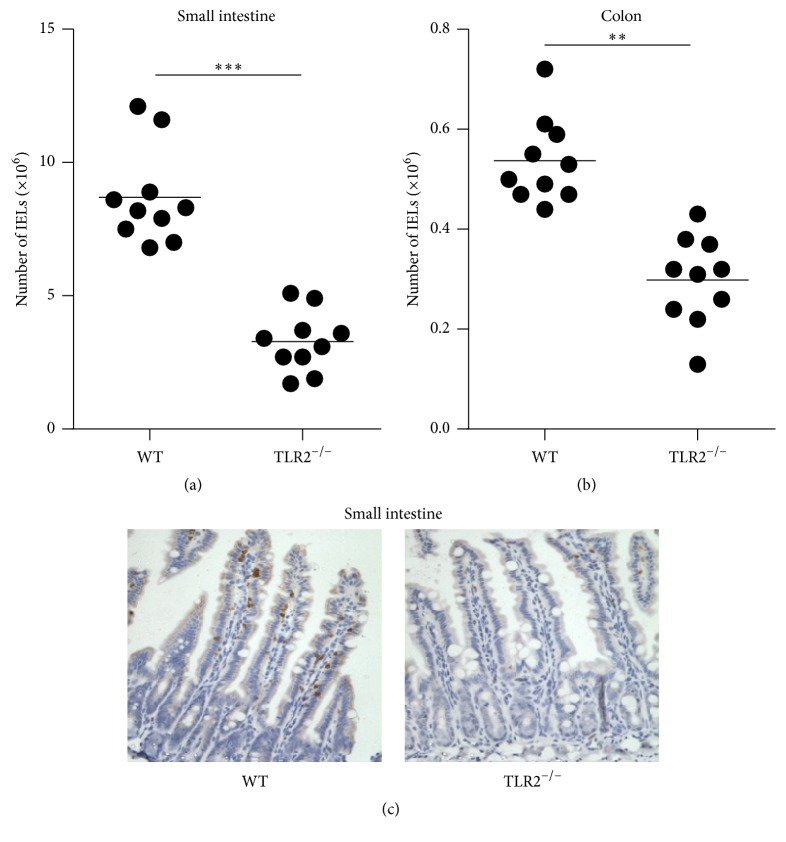
Changes in the number of intraepithelial lymphocytes (IELs) in TLR2^−/−^ mice. (a and b) The numbers of IELs in the small intestine (a) and colon (b) of TLR2^−/−^ mice and individual control mice. Horizontal bars indicate the mean. Ten mice per group from three independent experiments. (c) The IEL was detected through immunohistochemistry with CD3. Images are 400x. ^*∗∗*^ *P* < 0.01; ^*∗∗∗*^ *P* < 0.001.

To investigate whether TLR2 signaling has an impact on the homeostasis of IELs, we first examined the numbers of IELs in TLR2^−/−^ mice. The number of IELs was analyzed by flow cytometry. The total number of IELs in TLR2^−/−^ mice was reduced significantly in the small intestine and colon, respectively (Figures 1(a) and 1(b)). Immunohistochemistry was used to detect and localize the IELs, and most of these cells expressed CD3. In situ staining for CD3 confirmed that IELs were tightly interdigitated with IECs at the basolateral face (Figure 1(c)). Consistent with flow cytometry, there was a striking loss of CD3-positive cells in the small intestine in the absence of TLR2 compared with WT mice (Figure 1(c)).

### 3.2. Type b IELs Are Dramatically Decreased in TLR2^−/−^ Mice

**Figure 2:**
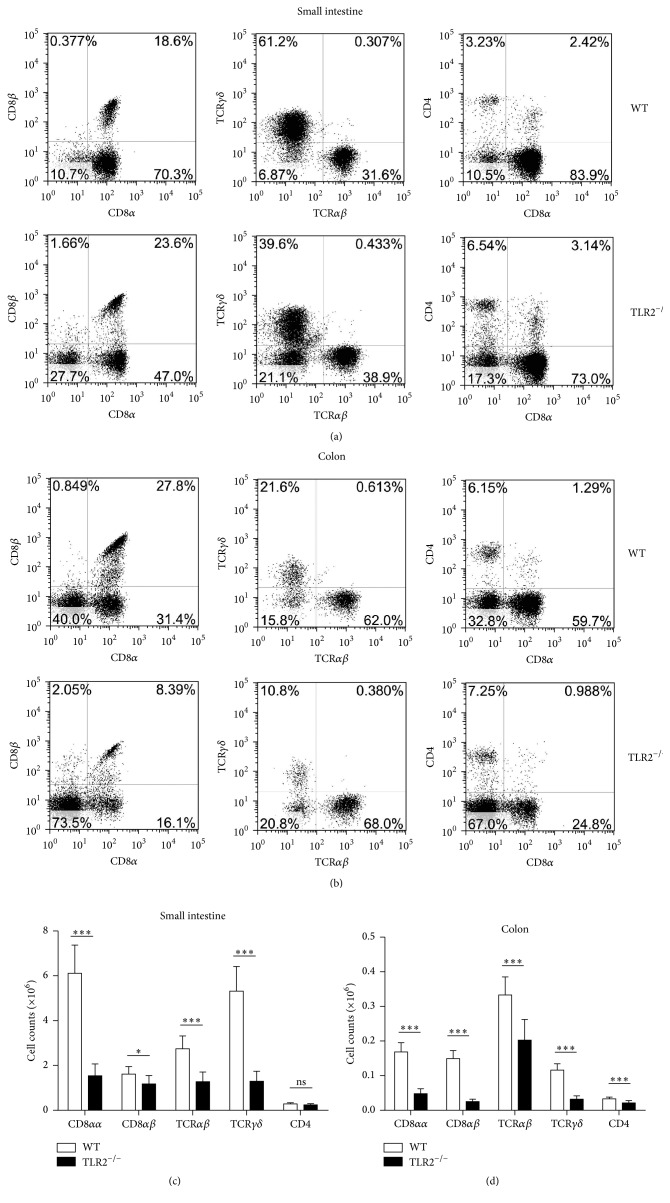
Changes in the phenotypes of intraepithelial lymphocyte (IEL) in TLR2^−/−^ mice. Cell populations are expressed as the percentage of gated cells with different cell phenotype markers. The data were obtained from CD45-positive cells. (a and b) The IELs from small intestine (a) or colon (b) of mice were stained as indicated. Expression of CD8*α* and CD8*β* chains on CD45^+^ IELs. Cells were stained with anti-CD45, anti-CD8*α*, and anti-CD8*β* mAbs and positively gated by CD45. Expression of TCR*αβ* and TCR*γδ* on CD45^+^ IELs. Cells were stained with anti-CD45, anti-TCR*αβ*, and anti-TCR*γδ* mAbs and positively gated by CD45. Expression of CD8*α* and CD4 on CD45^+^ IELs. Cells were stained with anti-CD45, anti-CD8*α*, and anti-CD4 mAbs and positively gated by CD45. (c and d) The absolute numbers of the indicated IEL subsets in the small intestine (c) or colon (d) of individual mice. The absolute number of each subset was calculated by multiplying total number of IELs by the percentage of each subset. Five mice per group. Representative of three experiments. ^*∗*^ *P* < 0.05; ^*∗∗∗*^ *P* < 0.001.

To better understand the changes occurring in the IELs after TLR2 knockout, a phenotype analysis was performed using flow cytometry. Typical results are presented in Figures 2(a) and 2(b), and the absolute numbers of IEL subsets are summarized in Figures 2(c) and 2(d). Analysis of small intestinal IELs in TLR2^−/−^ mice revealed that the unconventional CD8*αα* ^+^ (approximately 3.3-fold) and TCR*γδ* ^+^ (approximately 3.9-fold) IELs were dramatically reduced and the CD8*αβ* ^+^ IEL subset was significantly reduced (approximately 1.1-fold). There was no significant difference in the number of CD4^+^ IELs in small intestine between TLR2^−/−^ and WT mice, although their proportion relatively was increased in TLR2^−/−^ mice. Similar results were also observed in the colon of TLR2^−/−^ mice. However, CD4^+^ IELs from colon were significantly reduced in TLR2^−/−^ mice. These data thus indicate a critical and selective role for TLR2 in the homeostasis of IELs.

### 3.3. IELs Display Reduced Proliferation and Increased Apoptosis in TLR2^−/−^ Mice

**Figure 3:**
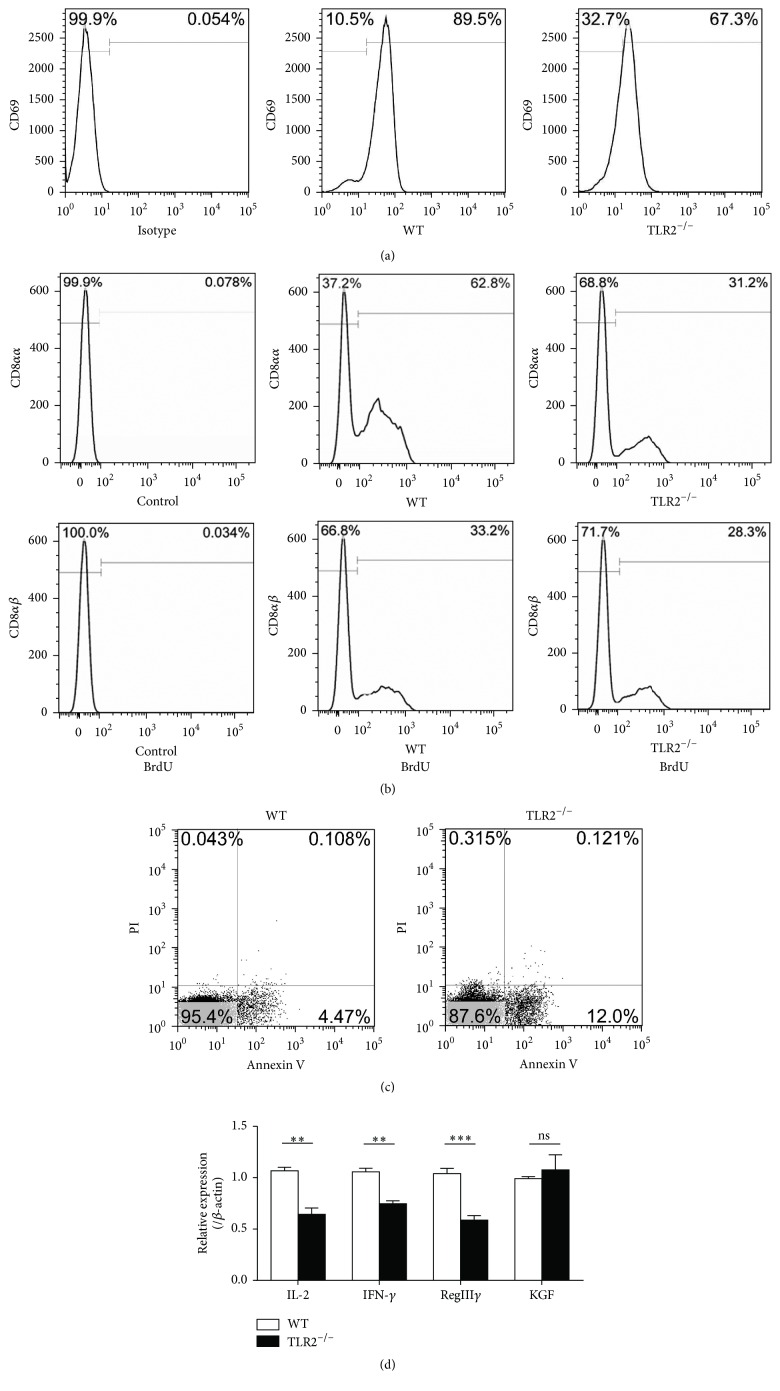
Alterations in IEL activation, proliferation, apoptosis, and cytokine mRNA expression after TLR2 knockout. (a) The surface expression of CD69 on IELs was detected by flow cytometry. (b) TLR2^−/−^ and wild-type mice were injected with BrdU twice per day. After 24 h, BrdU incorporation in the indicated IEL subsets was analyzed by flow cytometry. (c) Intestinal IELs were examined through flow cytometry for CD45 and apoptosis markers (FITC-Annexin V and PI). In the apoptosis map, FITC-Annexin V+/PI+ indicates late apoptosis, FITC-Annexin V+/PI− indicates early apoptosis, and FITC-Annexin V−/PI− indicates live cells. (d) Changes in the small intestinal IEL-derived cytokine mRNA measured using real-time RT-PCR. The results were expressed as the ratio of the number of copies of the gene of interest to the number of copies of the *β*-actin gene. Five mice per group. Representative of three experiments. ^*∗∗*^ *P* < 0.01; ^*∗∗∗*^ *P* < 0.001.

To investigate the contribution of TLR2-dependent proliferative and cell survival signals in IELs, we assessed their proliferative capacity by incorporation of BrdU in vivo. CD8*αα* ^+^ IELs showed poorer proliferation in TLR2^−/−^ mice, whereas the CD8*αβ* ^+^ IELs were normal (Figure 3(b)). We next examined whether TLR2 deletion affected the apoptosis of IELs. The residual IELs showed higher apoptosis in TLR2^−/−^ mice (Figure 3(c)). IELs are T cells that exist in some intermediate stage of activation, and CD69 expression might reflect the activated nature of IELs. Histogram showed a decrement in the expression of CD69 on the IELs from TLR2^−/−^ mice (Figure 3(a)). A panel of cytokines was selected to represent the activities responsible for IEL activation and intestine protection. Results are shown in Figure 3(d). IEL cytokine expressions were significantly altered after TLR2 knockout. The results suggest that IL-2, IFN-*γ*, and RegIII*γ* mRNA expression in TLR2^−/−^ IELs significantly decreased. However, KGF showed no significant difference between WT group and TLR2^−/−^ group (Figure 3(d)).

### 3.4. TLR2 Signaling in the IECs Maintains IL-15 Expression

**Figure 4:**
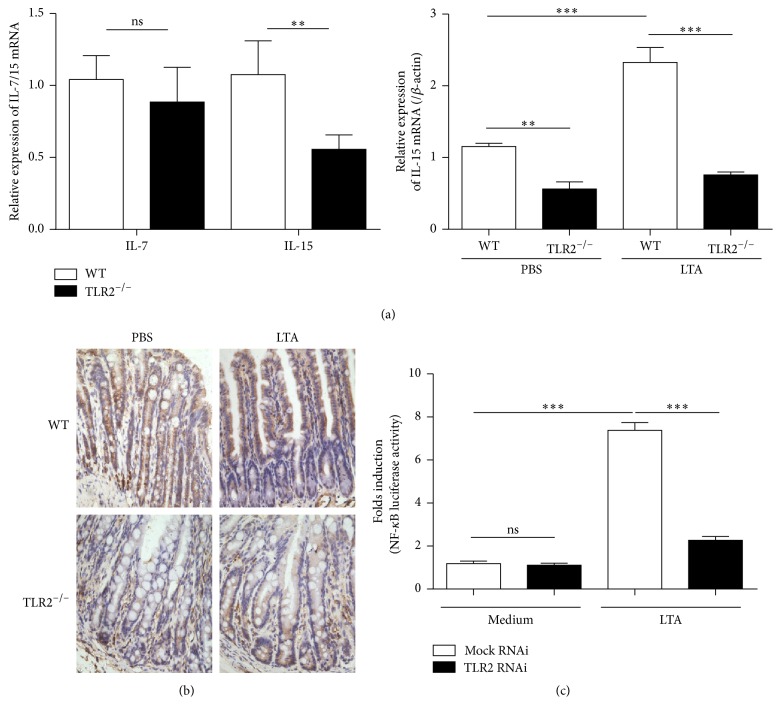
The effect of TLR2 knockout (down) on IL-15 expression in intestinal epithelial cells. (a) RT-PCR analysis of IL-7 and IL-15 expression in the jejunum sample. RNA was prepared from the samples of the TLR2^−/−^ mice and the wild-type mice. (b) Immunohistochemistry with IL-15 antibody on sections of small intestine from wild-type mice + PBS, wild-type mice + lipoteichoic acid (LTA), TLR2^−/−^ mice + PBS, and TLR2^−/−^ mice + LTA. Images are 400x. (c) SW480 cells were transfected with TLR2-siRNA or Mock and TK-RL and pBIIx-luc. At 24 h after transfection, the cells were stimulated with the indicated concentrations of LTA, and then both firefly and Renilla luciferase activities were determined using a dual-luciferase assay. The experiments were repeated three times, and all of the data were expressed as the mean ± SD. ^*∗∗*^ *P* < 0.01; ^*∗∗∗*^ *P* < 0.001.

We and others have previously reported that IL-7 and IL-15 play critical roles in the development of IELs [17]. We next examined IL-7 and IL-15 mRNA in the mucosa of jejunum by RT-PCR. Interestingly, the basal or LTA-induced expression of IL-15 in mucosa was decreased when TLR2 was absent (Figure 4(a)). However, the defect in IL-7 mRNA expression in TLR2^−/−^ mice was limited (Figure 4(a)). We also used immunohistochemistry to confirm the RT-PCR results suggesting that TLR2-driven signals regulated the expression of IL-15 in intestinal mucosa (Figure 4(b)). It is known that NF-*κ*B binding site is essential for transcriptional activation of the IL-15 gene. To test whether TLR2 agonist could activate signaling pathway in intestinal epithelial cell line, NF-*κ*B reporter activity was assessed after the TLR2 siRNA or mock siRNA-transfected SW480 was stimulated with LTA, respectively. As shown in Figure 4(c), LTA could induce high levels of NF-*κ*B reporter activity, whereas the response of TLR2 siRNA group to LTA was largely suppressed. Thus, our data strongly suggest that TLR2 signaling might maintain the expression of IL-15 in IEC via NF-*κ*B activation.

### 3.5. TLR2 Deficiency Increases Epithelial Immunopathology

**Figure 5:**
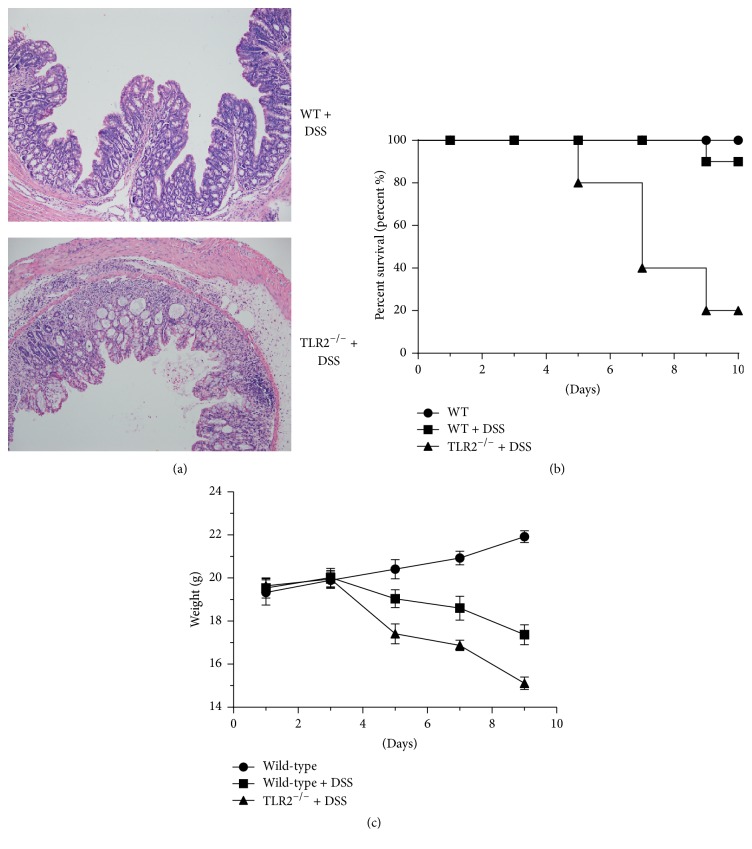
Histopathological characterization of DSS-induced colitis. (a) H&E staining of colons of 3% DSS-treated controls or TLR2-deficient mice. Images are 200x. (b) Survival rates were recorded. (c) Body weight was determined once every other day between day 1 and day 9. Ten mice per group from three independent experiments.

Loss of type b IELs in mice aggravated colitis in several animal models and resulted in impaired ability to repair damaged epithelia. In order to address the consequences of TLR2 deficiency for intestinal physiology, we employed DSS-induced colitis. The severity of the tissue damage in TLR2-deficient colons was illustrated in histological sections, showing diffuse lamina propria and increased destruction of colonic epithelium but reduced immune cell infiltration (Figure 5(a)). The overall mortality rate was 80% in DSS-TLR2^−/−^ mice versus 10% in DSS-WT mice (Figure 5(b)). Compared with WT controls, TLR2 knockout animals lost weight more rapidly, to a greater extent, and failed to gain weight during the course of the 9-day experiment (Figure 5(c)). These data suggest that TLR2 deficiency contributes to the impaired innate immune defense and high susceptibility to colitis in these mice.

## 4. Discussion

Triggering of PRRs in IECs leads to the expression of immune modulators, which have an impact on the regulation of the adjacent immune cells and are necessary to maintain intestinal mucosa barrier [18]. As IECs have long been recognized as a source of IL-15 in the intestines and are adjacent to IELs, this study determined whether the TLRs on IECs are associated with the homoeostasis of IELs through an IL-15-dependent manner. We found that TLR2-dependent signaling in IECs played an important role in development of IELs (especially CD8*αα* ^+^TCR*αβ* ^+^ and TCR*γδ* ^+^ IELs), and the signaling was essential in transcriptional activation of IL-15 via NK-*κ*B. Furthermore, we have shown that loss of IELs contributes to the high susceptibility of TLR2^−/−^ mice to DSS-induced colitis.

Although the study of TLR pathways in haematopoietic cells has mostly focused on their proinflammatory properties, their role in maintaining IELs homeostasis and immune tolerance has emerged as a major component of their function in IECs. In the mouse small intestine, the expression of TLR2 is found in 3 IEC lineages: enterocytes, Paneth cells, and enteroendocrine cells [19, 20]. In addition to regional localization, TLR2 expression in the intestine is also thought to be regulated spatially. TLR2 expression is found on both apical and basolateral sides of the follicle-associated epithelium but only the apical side of the villous IECs [7]. Taken together, the localization and expression of TLR2 suggest that the molecular patterns that are actually recognized by TLR2 in the intestine are generally not from pathogens but from the commensal flora. Thus, we focus on the role of TLR2 in shaping the repertoire of IELs in the small intestine and colon. In this study, we showed that the total number of IELs in TLR2^−/−^ mice was reduced significantly in the small intestine and colon as compared with WT mice. Moreover, TLR2^−/−^ mice showed dramatically reduced numbers of CD8*αα* ^+^TCR*αβ* ^+^ and TCR*γδ* ^+^, similar to the proportions found in IL-15^−/−^ mice [4]. However, CD8*αβ* ^+^ IELs were moderately reduced in TLR2^−/−^ mice. These results suggest that type b IELs are more sensitive to TLR2 deficiency. Interestingly, Shin and Iwasaki found that CD8*αβ* ^+^ tissue-resident memory T cells (belong to type a IEL) expressed less IL-15Rb [21]. Based on these facts, we speculated that type b IELs may express more IL-15R compared to type a IELs. Moreover, Yu et al. found that the CD8*αα* ^+^TCR*αβ* ^+^ and TCR*γδ* ^+^ IELs were selectively decreased in myeloid differentiation factor 88- (MyD88-) deficient mice [22]. And we all know that the MyD88-dependent pathway is utilized by all TLRs except TLR3. Here, we identify previously unappreciated TLR2-mediated signaling necessary for development or maintenance of IEL populations. Consistent with an immune-modulator role of TLR, ~1.8% of all murine transcripts were affected significantly by TLR2 deletion, a number of which correlated with immune processes changes [23]. However, our data showed that the CD8*αα* ^+^TCR*αβ* ^+^ and CD8*αα* ^+^TCR*γδ* ^+^ IEL subsets did not completely disappear in TLR2^−/−^ mice. This may be because the expression of IL-15 in intestinal mucosa was reduced but not completely lost in TLR2^−/−^ mice, which is consistent with our immunohistochemistry findings (Figure 4(b)). Alternatively, the IELs may depend for their development and maintenance on factors other than IL-15, such as IL-2 and IL-7 [24, 25].

Homeostasis of peripheral lymphocytes is regulated by proliferation and apoptosis. Lai et al. reported that IL-15 did not affect IEL development in the thymus but regulated homeostasis of IELs in the intestine [26]. It has been shown that intestinal IL-15 supports CD8*αα* ^+^ IELs survival through the activation of the PI3K-Akt-ERK pathway to upregulate Bcl-2 and Mcl-1 [27]. In the present study, we showed that the residual IELs displayed increased apoptosis in TLR2^−/−^ mice. In addition to its effects on IEL survival, IL-15 is also known for the ability to enhance proliferation of isolated IELs in vitro [28]. To investigate the contribution of TLR2-dependent proliferative signals in IELs, we assessed their proliferative capacity by incorporation of BrdU in vivo. The CD8*αα* ^+^ IELs showed poorer proliferation in TLR2^−/−^ mice, whereas the CD8*αβ* ^+^ IELs held the normal capacity. Therefore, these results suggest that IEC contributes to the maintenance of IELs at least partly via TLR2-dependent IL-15 production. Unlike T cells in other peripheral immune compartments, IELs express some markers of activated T cells, such as the CT antigen (in mice) and CD69, suggesting that they constitute a large population of “partially activated” effector cells [29]. Moreover, the majority of IELs can secrete epithelial growth factors and produce Th1 cytokines and antimicrobial peptides, such as RegIII*γ* [30]. In this study, TLR2^−/−^ mice had a lower level of IEL activation than WT mice. Additionally, the residual IELs in TLR2^−/−^ mice expressed a reduced level of IFN-*γ*, IL-2, and RegIII*γ*. Consistent with our study, other researchers have found that commensal bacteria are required for DSS-induced expression of proinflammatory cytokines and the antibacterial lectin RegIII*γ* in IELs [31]. Furthermore, TCR*γδ* ^+^ IELs activation was found to be dependent on epithelial cell-intrinsic MyD88 [22]. Thus, the above lines of evidence indicate that IECs supply microbe-dependent cues to IELs via TLRs.

IL-15 was found to be produced only by limited populations of cells, such as dendritic cells and epithelial cells, but not by activated T cells. And perhaps, more importantly, Ma et al. demonstrated that transpresentation of IL-15 by IECs alone is completely sufficient to direct the IL-15-mediated development of CD8*αα* ^+^ IELs [4]. We thus investigated whether TLR2 signaling affected the production of IL-15 by IECs. Immunohistochemistry results showed that IL-15 is mainly expressed in intestinal epithelium in the basal condition. As expected, the basal expression of IL-15 in epithelium was decreased when TLR2 was absent. It is known that NF-*κ*B binding site is essential for transcriptional activation of the IL-15 gene [32]. In this regard, we observed that the TLR2 siRNA decreased the NF-*κ*B gene reporter activity in response to LTA (TLR2 agonist) in intestinal epithelial cell line SW480. Therefore, as discussed above, it is now apparent that TLR2-MyD88-dependent signaling is important for transcriptional activation of IL-15 gene in IEC and consequently for development and/or maintenance of IEL populations. More researches need to be done for confirming this pathway in IEC and defining the commensal bacterial species that elicit this effect.

Although the etiology of IBD is poorly understood, increasing evidence suggests that IBD is caused in genetically susceptible individuals by a dysregulated mucosal immune response to intestinal microorganisms. Advances risen from genome-wide association studies (GWAS) and immunological studies have recently moved the focus of IBD pathogenesis onto mucosal innate immune responses, such as epithelial barrier integrity, innate microbial sensing, autophagy, and unfolded protein response, as central pathogenic pathways in IBD [33]. IECs from patients with IBD have higher expression of TLRs, especially TLR4, and similar or lower expression of TLR2, TLR3, TLR5, and TLR9 than IECs from control individuals [7]. Systemic administration of the TLR2 ligand tripalmitoyl-S-glyceryl cysteine-serine_4_-lysine (Pam3CSK4) protects against DSS-induced colitis [34]. In this study, TLR2^−/−^ mice were susceptible to the colitis induced by DSS. The above results suggest that IELs dysregulation caused by loss of TLR2 may favor the onset of colitis. Indeed, data from our previous study suggest that loss of CD8*αα* ^+^TCR*αβ* ^+^ and TCR*γδ* ^+^ aggravates colitis in the mouse model [35]. However, we also cannot ignore the emerging role of TLR2 in protecting TJ-associated integrity and enhancing transepithelial resistance of the enterocyte barrier [34].

## 5. Conclusion

Collectively, TLR2-dependent signaling is important in keeping the number of the IEL populations in the basal condition. Mice with TLR2 deletion lacked IELs, especially the CD8*αα* ^+^ IELs (type b IELs), in the small intestine and colon. The residual IELs displayed reduced proliferation and activation and increased apoptosis in TLR2^−/−^ mice, accompanied with impaired IL-15 expression by IEC. Moreover, our results also indicate that deficiency of TLR2 contributes to the high susceptibility of mice to DSS-induced colitis. These results suggest that TLR2-dependent signaling for IL-15 production from interaction between commensal bacteria and IEC plays an important role in maintenance of homeostasis of IEL.
